# Fenofibrate ameliorated atorvastatin and piperine-induced ROS mediated reproductive toxicity in male Wistar rats

**DOI:** 10.1016/j.toxrep.2024.101861

**Published:** 2024-12-10

**Authors:** Sanjib Ghosh, Sweata Sarkar, Maharaj Biswas

**Affiliations:** a Endocrinology Laboratory, Department of Zoology, University of Kalyani, West Bengal 741235, India; b Department of Zoology (PG Studies), Rishi Bankim Chandra College, West Bengal 743165, India

**Keywords:** Atorvastatin, Piperine, Fenofibrate, ROS, Reproductive toxicity

## Abstract

Atorvastatin and fenofibrate are well-known lipid-lowering drugs. Atorvastatin acts by reducing the production of cholesterol through the inhibition of the 3-hydroxy-3-methyl-glutaryl-coenzyme A reductase (HMG Co-A reductase) enzyme, whereas fenofibrate is a PPAR-α agonist. Piperine is an alkaloid mostly found in black pepper fruits. The present study was planned to evaluate the activities of atorvastatin, fenofibrate, and piperine on the male reproductive system. A total of 35 male Wistar rats were obtained for the experiment. Rats were randomly divided into 7 groups, each group with 5 rats. The experiment was run for 28 days. Group I rat got normal meals for 28 days; Group II received atorvastatin (08 mg/kg/day); Group III received piperine (10 mg/kg/day); and Group IV received fenofibrate (20 mg/kg/day). Group V received atorvastatin (8 mg/kg/day) and piperine (10 mg/kg/day); Group VI received piperine (10 mg/kg/day) and fenofibrate (20 mg/kg/day). VII received fenofibrate (20 mg/kg bw/day) and atorvastatin (8 mg/kg/day). After sacrifice, serum and testicular cholesterol and testosterone levels assessed by ELISA, ROS generation analysed by using flow cytometry, MDA, SOD, and catalase were measured. Histological, sperm-parameter analysis, and spermatogenic evaluations were also done. Activities of atorvastatin and piperine revealed reproductive toxicity upon treatment. Fenofibrate treatment, along with atorvastatin and piperine, showed protective effects. In conclusion, atorvastatin and piperine affected reproductive potential, whereas fenofibrate might have protective efficacy against atorvastatin and piperine-induced reproductive toxicity.

## Highlights

• Both atorvastatin and piperine affected sperm-parameters.
• Co-administration of atorvastatin and piperine distorted spermatogenesis in seminiferous tubules.
• Fenofibrate treatment showed protective effects upon atorvastatin and piperine induced reproductive toxicity.

## Graphical Abstract

**Figure:**
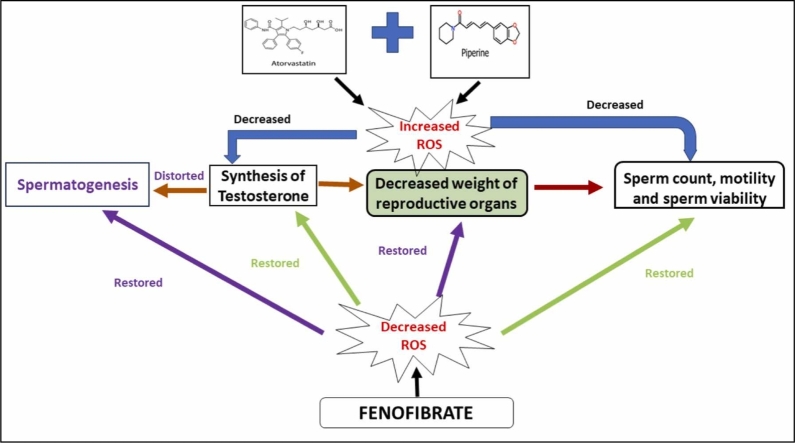

## 1 Introduction

**Fig. 1:**
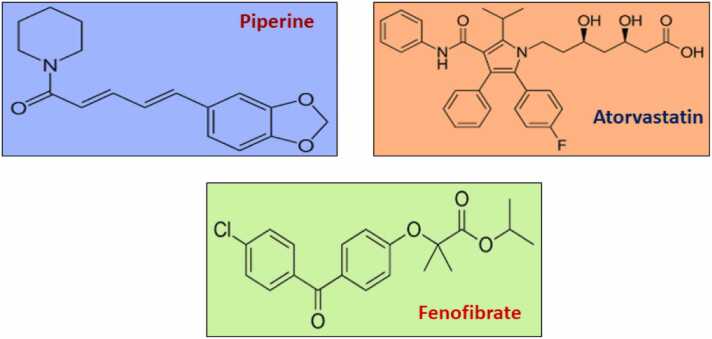
Chemical structure of Atorvastatin, Piperine and Fenofibrate.

It is estimated that approximately 15 % of couples who are attempting to conceive are clinically infertile, and male-factor infertility is involved in fifty percent of those cases [1]. In addition, almost 25 % of male infertility patients exhibit abnormal semen tests without any identifiable explanation [1]. The growing prevalence of male infertility is poised to become a prominent reproductive issue in the current century. The reproductive potential of males is susceptible to a multitude of environmental and lifestyle factors [2]. Several therapeutic drugs have been reported to impair male fertility [2], [3]. Statins are drugs commonly used to decrease lipid levels to mitigate the risk of developing cardiovascular disorders [4]. Statins work by reducing the production of cholesterol through the inhibition of 3-hydroxy-3-methyl-glutaryl-coenzyme A reductase (HMG Co-A reductase) enzyme [5]. Atorvastatin or ATR (Fig. 1) is one of the most important and frequently prescribed synthetic statins [6]. Cholesterol plays a crucial role in male reproductive potential as it directly affects steroidogenesis, spermatogenesis, and fertilization [7]. Cholesterol is the precursor molecule for testosterone biosynthesis and testosterone is needed for proliferation and differentiation of spermatogonia [8]. Cholesterol is directly linked to sperm motility and capacitation [9]. Impaired lipid metabolism is strongly associated with male infertility [10]. Numerous researchers have long argued that the consumption of atorvastatin negatively affects male fertility [11], [12], [13]. Cholesterol-lowering medications have been hypothesised to impact steroid synthesis in males due to their therapeutic effects on cholesterol [14], [15]. Recent studies conducted on both human and rat skeletal muscle have shown that statin treatment leads to an increase in the generation of reactive oxygen species [16].

Piperine or PIP (Fig. 1) is a predominant secondary metabolite found in black pepper (*Piper nigrum* L.). In many parts of the world, black pepper is a highly popular spice [17]. In humans as well as animals, papering can increase the bioavailability of a variety of drugs and nutrients. [18]. Mishra and Singh reported antifertility and anti-spermatogenic effects of piperine and black pepper in male rats characterized by degenerative changes on seminiferous tubules, epididymis, and sperm parameters [19]. Ethanol extract of black pepper fruit have seen to decrease the number of primary spermatocyte and sperm motility in experimental rat [20]. Classical work of Malini et al. (1999) also reported anti-spermatogenic effects of piperine [21]. Piperine is also reported to initiate apoptosis in testicular tissues [22]. Chinta G et al. reported that piperine disrupted the testicular antioxidant system by promoting the ROS production [23].

Both ATR and PIP were reported to generate ROS in various tissues [16], [23] including testis. Elevated levels of reactive oxygen species (ROS) cause adverse effects on the cells in the testes and result in impaired sperm production [24]. Oxidative stress is an established cause of male infertility [25]. The plasma membrane of spermatozoa is made up of polyunsaturated fatty acids (PUFAs) that make them susceptible to lipid peroxidation and damage caused by ROS [26]. Oxidative stress affects chromatin integrity of spermatozoa [27] and resulted in high occurrence of apoptosis in testicular tissues [28]. It was also claimed by several researchers that ROS can reduce male fertility by affecting male sex hormone levels [29], [30].

Fenofibrate or FEN (Fig. 1), a fabric acid derivative, is used as an antihyperlipidemic medication to treat dyslipidaemia by acting as a ligand for peroxisome proliferator-activated receptors-α (PPAR-α) [31]. In testis, PPARα is mainly expressed in Leydig cells and at lower levels in Sertoli cells [32]. In testicular tissue, the antioxidant defence system (ADS) is comprised of different enzymatic elements (such as superoxide dismutase and catalase) as well as nonenzymatic elements like glutathione [33]. PPARα is a target for fibrates, PPARα stimulation increase the genes expression of superoxide dismutase or SOD [34]. PPAR-a agonists such as fenofibrate have been studied to have antioxidant properties [35]. Several researchers have claimed that PPARα have direct connection with male reproductive system [36], [37].

## 2 Materials and methods

### 2.1 Drugs and chemicals

Piperine (97 %, P49007) is purchased from Sigma-Aldrich (Germany). The atorvastatin and fenofibrate used in this experiment were purchased from Lupin Ltd. (India) and Cipla Ltd. respectively. All other chemicals and reagents used in this experiment were extremely pure and of analytical quality. PBS was purchased from SRL (India).

### 2.2 Animals

The investigation was conducted on adult, healthy male Wistar rats with an average body weight ranging from 200 to 220 g. The animals were purchased from the Centre for Laboratory Animal Research and Training, Kalyani, West Bengal, India. The rats were accustomed to the laboratory conditions for a period of 2 weeks. The rats were kept under standardized laboratory conditions, with a room temperature of 24–26°C, a relative humidity of 50–70 %, and a 12-hour light/dark cycle. The IEAC, University of Kalyani, has granted approval (892/GO/Re/S/01/CPCSEA) for all experimental procedures conducted on the animals.

### 2.3 In vivo experiment design

A total of 35 male Wistar rats were obtained for the experiment. Rats were randomly divided into 7 groups, each group with 5 rats. The experiment was run for 28 days.

**Group 1(Control)**: rats received normal diets daily for 28 days,

**Group II (Ta)**: rats were gavaged atorvastatin (08 mg/kg bw/ day) for 28 consecutive days;

**Group III (Tp)**: given piperine (10 mg/kg bw/day) for 28 consecutive days.

**Group IV (Tf)**: given fenofibrate (20 mg/kg bw/day) for 28 consecutive days.

**Group V (Tap)**: were co-administered with atorvastatin (8 mg/kg bw/day) and piperine (10 mg/kg bw/day) for 28 consecutive days.

**Group VI (Tfp)**: were co-administered with piperine (10 mg/kg bw/day) and fenofibrate (20 mg/kg/day) for 28 consecutive days

**Group VII (Taf)**: were co-administered with fenofibrate (20 mg/kg bw/day) and atorvastatin (8 mg/kg/day) for 28 consecutive days.

### 2.4 Sample collection

Upon completion of the experiment, all animals were administered intraperitoneal anaesthesia with a dosage of 80 mg/kg ketamine and 10 mg/kg xylazine. Following the euthanasia of the animals through sacrifice, both testes were removed and preserved at a temperature of −20 °C for further biochemical examination.

### 2.5 Sperm count, motility, and sperm viability analysis

Following dissection, the epididymis was thoroughly cleansed and the caudal area was subsequently divided into smaller sections. Small fragments of the cauda epididymis were placed in a petri dish with 2 ml of a 1X PBS solution. The cauda was punctured with a needle to release the spermatozoa into the liquid medium. The petri dish was incubated at a temperature of 37 ºC with 5 % CO_2_ for a duration of 10 minutes to allow the sperm to exit the tubules by swimming. The suspension in the petri dish is subsequently analysed to quantify sperm count, sperm motility, and sperm viability under a ZEISS Primo Star light microscope at 40X magnification [38].

Using an upgraded Neubauer hemacytometer, the number of sperm was ascertained [38]. The epididymal sperm suspension was diluted in a 1:5 ratio with PBS medium to examine the sperm concentration. The diluted sperm sample was then fed into the two central grids of a Neubauer’s counting chamber. Under a light microscope (ZEISS Primo Star) with a 40 × magnification, the quantity of sperm cells was counted from both chambers and averaged.

To assess the motility of the sperm, ten microliters of the suspension were placed on a sterile, previously heated slide and covered with a cover slip. By using a light microscope equipped with a heated stage, a minimum of ten tiny areas were examined under increased magnification. Following isolation, 200 sperm cells were examined under a ZEISS Primo Star microscope for a duration of two to four minutes.

The viability of the sperm was assessed using a combination of Eosin-Y (0.05 %) and nigrosin [38]. The sperm solution was combined with Eosin-Y-Nigrosin in a 1:1 ratio, with each component measuring 20 μL. By employing this method, cells that have undergone alterations in their plasma membrane exhibit a pink coloration, whereas spermatozoa with an intact plasma membrane retain their original colour. The viability of spermatozoa was assessed by calculating the ratio of unstained spermatozoa/pink spermatozoa and expressing the results as a percentage.

### 2.6 Haematoxylin-Eosin staining of testis

The right testis from each rat was fixed in Bouin’s fluid, dehydrated in graded series of ethanol, and washed in xylene. The tissues were embedded in paraffin wax to form paraffin blocks and then the paraffin sections of 5μm thick were stained with haematoxylin and eosin. The histomorphometric changes in seminiferous tubules were observed under a Zeiss Axiolab upright microscope.

Johnsen’s scoring (JS) method was used for assessing testicular tissue damage and status of spermatogenic cells [39]. JS utilizes a scoring from 1 to 10 according to the presence or absence of spermatogenic (spermatozoa, spermatids, spermatocytes, spermatogonia) cells and Sertoli cells. JS was done using 25 randomly selected seminiferous tubules (ST) from each experimental groups under a Zeiss Axiolab upright microscope at 40X magnification. High JS (10−6) signifies normal or good status of spermatogenesis whereas low JS (4−1) indicates abnormality in spermatogenesis (Table 1).

**Table 1:** Histological grading of seminiferous tubules using Johnsen’s Scoring System.

Score	Description
10	Complete spermatogenesis with numerous spermatozoa in seminiferous tubule lumen. Germinal epithelium is regular.
9	Many spermatozoa with distorted germinal epithelium.
8	A few spermatozoa per tubule.
7	No spermatozoa but many spermatids in seminiferous tubules.
6	No spermatozoa and a few spermatids present in the tubules.
5	No spermatozoa or spermatids, many spermatocytes present.
4	No spermatozoa or spermatids, few spermatocytes present.
3	Only spermatogonia were present.
2	No germinal cells, Sertoli cells only.
1	No cells in seminiferous tubules.

### 2.7 Serum and testicular cholesterol assessment

Serum total cholesterol was measured by colorimetric methods using lipid profile test kit according to the manufacturer’s instructions (Robonik Pvt. Ltd. India).

In a screw-capped centrifuge tube, 0.1 ml of the supernatant was combined with 4.9 ml of ferric chloride solution (50 mg FeCl3 6H2O, dissolved in 100 ml acetic acid) after testicular tissue (50 mg/ml) was homogenised in an ether-alcohol mixture (1:3) and centrifuged at 3000 rpm for 10 min. The mixture was then left to stand for 15 minutes. After centrifuging the mixer for ten minutes at 3000 rpm, 1.5 ml of concentrated H2SO4 was added to 2.5 ml of the clear supernatant, and the mixture was left to develop colour for approximately half an hour at room temperature. Using a spectrophotometer, the colour intensity of the unknown and standard was measured against a blank at 560 nm using the Zlatkis et al. (1953) method. The outcome was given as mg/gm tissue [40].

### 2.8 ELISA for serum and testicular testosterone quantification

Serum testosterone concentration of control and six treatment groups were measured by using ELISA testosterone kit according to the manufacturer’s instructions (Accu-bind, Monobind Inc. CA, USA). About 100 mg of testicular tissue was homogenized in 1 ml of PBS buffer (PH – 7.4). Homogenates were centrifuged at 10,000 rpm for10 min. [41] and the resultant supernatant used immediately for determination of testosterone by ELISA Method (AcccuBind™ ELISA). Testicular testosterone content was calculated as ng / ml / mg of tissue.

### 2.9 Assessment of reactive oxygen species (ROS) generation in epididymal spermatozoa by DCFDA method

The measurement of reactive oxygen species (ROS) was conducted using a modified fluorometric assay [42], with 29, 79-dichlorofluorescin diacetate (DCFH-DA) serving as the probe. In summary, the cauda epididymal spermatozoa were preincubated for 15 minutes at room temperature with DCFH-DA (10 mmol). This allowed the probe to be integrated into any vesicles bound to the membrane, and the diacetate group was cleaved by esterases. The transformation of DCFH into the fluorescent compound dichlorofluorescein (DCF) was assessed after an incubation period of 25–30 minutes. This was done by measuring the fluorescence using a spectrophotometer with an excitation at 485 nm and emission at 530 nm.

### 2.10 Lipid peroxidation assay to detect oxidative stress marker MDA in testis

The lipid peroxidation experiment was conducted following the modified method of Iqbal et al.1996 [43]. The experiment was conducted in duplicate using 200 μl of homogenates combined with 0.58 ml phosphate buffer, 200 μl ascorbic acid, and 20 μl ferric chloride. The blank, which served as a control, contained distilled water instead of homogenate. The solution was subjected to a mechanical water bath at a temperature of 37°C for a duration of 1 hour. The reaction process was halted by adding 1 ml of TCA solution, followed by the addition of 1 ml of TBARS. The mixture was then placed in a boiling water bath for 20 minutes. After being cooled in a crushed ice bath for approximately 3–4 minutes, the solution was then subjected to additional centrifugation at 3000 rpm for 10 minutes. The liquid portion was collected and the intensity of light absorption was determined at a wavelength of 535 nm using a spectrophotometer, comparing it to a solution without the reagent. The result of the assay was expressed in nMTBARS/min/mg tissue, using molar extinction coefficient 1.5X105M^−1^cm^−1^.

### 2.11 Assessment of antioxidant enzyme activities (SOD and catalase) in testis

A method developed by Beauchamp and Fridovich [44] was used to determine SOD activities. This method involves the inhibition of nitroblue tetrazolium reduction by SOD using the NBT compound. The absorbance measurements were subsequently converted into SOD activity units per ml or per mg protein. Each unit of SOD activity corresponded to the amount of SOD required to decrease the background rate of nitroblue tetrazolium reduction by 50 %.

The method of determining catalase activity was based on Sinha's research in 1972 [45]. In a nutshell, a solution was prepared by combining 2 ml of hydrogen peroxide (800 μ moles), 2.5 ml of 0.01 M phosphate buffer (pH 7.0), and 0.5 ml of diluted sample (1:50) at a rapid pace and at a temperature of 25 °C. Subsequently, 1 ml of the reaction mixture was taken out and quickly mixed with 2 ml of dichromate/acetic acid solution at 1-minute intervals to determine the remaining H_2_O_2_ in the solution. The chromic acetate produced was measured at 570 nm, while the remaining H_2_O_2_ was estimated by extrapolating from the standard curve for hydrogen peroxide. The catalase activity was measured in terms of the amount of H_2_O_2_ consumed per minute per milligram of protein.

### 2.12 Statistical analysis

The calculation was done by GraphPad Prism Software. All the experiments were done in duplicates. The data were expressed as Mean ± SEM; n = 5 per treatment. Statistical analysis was done by one way ANOVA using Tukey’s post-hoc test. Significant differences were considered at *P* < 0.05 and *P* < 0.01.

## 3 Results

### 3.1 Changes in body weight and reproductive organ weights of male rats

**Fig. 2:**
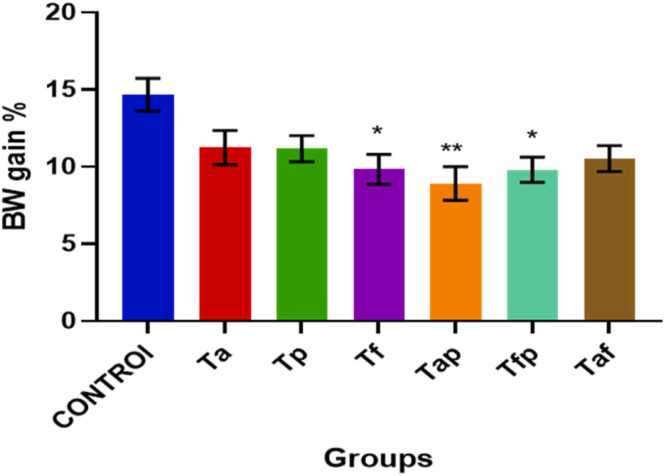
Changes in Body weight gain % of control group and treatment groups. The values were expressed as Mean ± SEM (n = 5). * p < 0.05 and **p < 0.01.

Changes in body weight gain percentage is depicted in Fig. 2. It is clearly seen that all treatment groups have decreased body weight percentage when compared to control group. On the other hand, significant decline in body weight percentage were found in Tf (p < 0.05), Tap (p < 0.01) and Tfp (p < 0.05) groups (Fig. 2).

Significant reduction in relative weight of testis were observed in Ta (p < 0.05), Tp (p < 0.01), Tap (p < 0.05) and Tfp (p < 0.05) groups when compared to the control group, whereas Tf and Taf group showed no significant changes when compared to the control group (Table 2).

**Table 2:** Relative weight of reproductive organs of Control and treatment groups.

	Relative weight of reproductive organs			
	Testis	Epididymis	Ventral Prostate	Seminal Vesicle
Control	1.304 ± 0.0149	0.573 ± 0.0098	0.128 ± 0.0031	0.323 ± 0.0079
Ta	1.198 ± 0.0193 *	0.511 ± 0.0048 *	0.112 ± 0.0039	0.288 ± 0.0082 *
Tp	1.082 ± 0.0182 * *	0.491 ± 0.0082 *	0.103 ± 0.0029 *	0.285 ± 0.0045 *
Tf	1.268 ± 0.0179	0.556 ± 0.0153	0.129 ± 0.0041	0.316 ± 0.0062
Tap	1.077 ± 0.0165 * *	0.446 ± 0.0092 * *	0.102 ± 0.0041 * *	0.278 ± 0.0080 *
Tfp	1.213 ± 0.0074 *	0.566 ± 0.0111	0.124 ± 0.0030	0.318 ± 0.0023
Taf	1.296 ± 0.0123	0.563 ± 0.0126	0.124 ± 0.0028	0.307 ± 0.0053

Changes in Body weight gain % of control group and treatment groups. The values were expressed as Mean ± SEM (n = 5). * p < 0.05 and * *p < 0.01.

In case of epididymis, Ta (p < 0.05), Tp (p < 0.05) and Tap (p < 0.01) groups showed significant reduction in relative weight in comparison with that of control group. Only Tp (p < 0.05) and Tap (p < 0.01) groups exhibited significant decrease in relative weight of ventral prostate gland when compared to control group and rest of the treatment groups (Ta, Tf, Tfp and Taf) showed no significant changes. Relative weight of seminal vesicle in Ta (p < 0.05), Tp (p < 0.05) and Tap (p < 0.05) groups reduced significantly when compared to that of control group (Table 2).

### 3.2 Effects on sperm count, sperm motility and sperm viability

Sperm count of Ta (p < 0.05), Tp (p < 0.01) and Tap (p < 0.01) groups significantly declined but that of Tf, Tfp and Taf groups showed no significant changes in comparison with the control group (Table 3). Percentage of sperm viability decreased significantly in Ta (p < 0.01), Tp (p < 0.01) and Tap (p < 0.01) groups but no significant changes were found in Tf, Tfp and Taf groups. In case of motility analysis, reduction in sperm motility was highly significant in Ta, Tp and Tap groups when compared to the control group. There was no significant change in sperm motility of Tf, Tfp and Taf groups (Table 3).

**Table 3:** Sperm count, viability and motility in control and experimental groups.

	Sperm Count (X10^6^/ml)	Viability (%)	Motility (%)
Control	29.4 ± 1.33	87.6 ± 2.54	77.8 ± 2.60
Ta	21.4 ± 0.93 *	71.2 ± 2.92 * *	60.6 ± 2.14 * *
Tp	20.0 ± 1.58 * *	59.6 ± 2.84 * *	51.6 ± 2.20 * *
Tf	26.0 ± 1.79	82.2 ± 1.98	73.2 ± 2.96
Tap	17.6 ± 1.44 * *	53.6 ± 3.08 * *	47.4 ± 1.29 * *
Tfp	25.4 ± 2.11	79.2 ± 3.20	69.4 ± 1.96
Taf	23.0 ± 1.82	83.8 ± 3.18	74.2 ± 1.77

Changes in Body weight gain % of control group and treatment groups. The values were expressed as Mean ± SEM (n = 5). * p < 0.05 and * *p < 0.01.

### 3.3 Effects on histomorphometry of seminiferous tubules

**Fig. 3:**
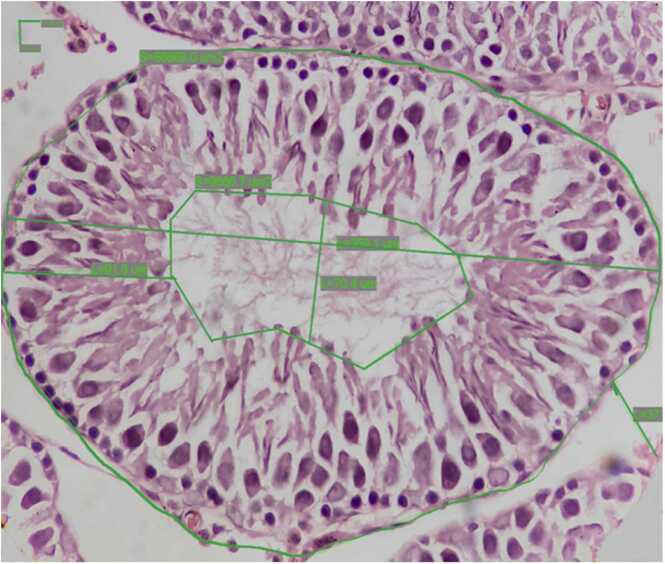
Photomicrograph showing measurement of STD, TL, GEH, and IS. STD- Seminiferous tubule diameter, TL- tubular lumen diameter, GEH- Germinal Epithelial height, IS- Interstitial space. Magnification 40x.

Fig. 3 depicted measurement of different types of histomorphometric features of a seminiferous tubule. A significant reduction in diameter of seminiferous tubules of Ta (p < 0.05) and Tap (p < 0.05) groups were observed (Table 4). Diameter of seminiferous tubule lumen increased in a significant manner in Ta (p < 0.05), Tp (p < 0.01) and Tap (p < 0.01) groups in comparison with the control group. Significant decrease in germinal epithelial height of Ta (p < 0.05), Tp (p < 0.05) and Tap (p < 0.05) groups were seen whereas the GEH of other treatment groups remained unchanged in comparison with the control group. Interstitial space diameter in all the treatment groups increased significantly when compared to the control group (Table 4).

**Table 4:** Showing changes in different types of histomorphometric measurements in control and experimental groups.

	STD (µm)	TL (µm)	GEH (µm)	IS (µm)
**Control**	308 ± 7.36	97.8 ± 4.61	101 ± 3.77	5.6 ± 0.68
**Ta**	259 ± 6.19 * *	114.4 ± 4.31 *	92.2 ± 3.44 *	13.2 ± 1.39 * *
**Tp**	305.2 ± 5.56	134.2 ± 4.65 * *	92.8 ± 3.12 *	16.6 ± 1.50 * *
**Tf**	305.2 ± 5.56	103.6 ± 4.07	110.6 ± 4.58	10.6 ± 0.93 * *
**Tap**	266.2 ± 6.67 *	133.8 ± 4.74 * *	92.6 ± 3.01 *	19.6 ± 2.18 * *
**Tfp**	315.4 ± 5.78	101.8 ± 4.15	110.4 ± 4.53	8 ± 1.00 *
**Taf**	311.6 ± 7.20	103.8 ± 3.10	107.4 ± 4.20	10 ± 0.71 * *

STD- Seminiferous tubule diameter, TL- tubular lumen diameter, GEH- Germinal Epithelial height, IS- Interstitial space. Changes in Body weight gain % of control group and treatment groups. The values were expressed as Mean ± SEM (n = 5). * p < 0.05 and * *p < 0.01.

### 3.4 Effects on histopathology of seminiferous tubules

**Fig. 4:**
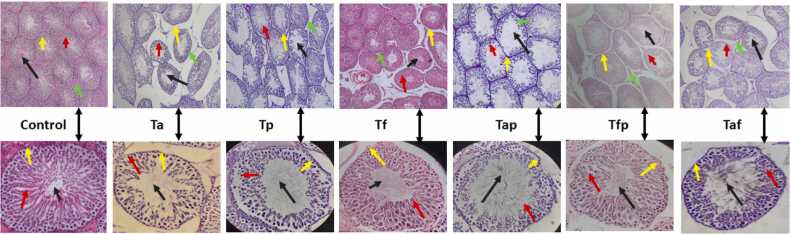
Photomicrograph of seminiferous tubules at 10x and 40x magnification. Yellow arrows denoted changes in basement membrane, red arrows denoted changes in Germinal epithelial layer, black arrows denoted changes in lumen shape and size and green arrows denoted changes in interstitial space.

Basement membrane of Ta, Tp and Tap groups were found to be mostly affected whereas basement membrane of seminiferous tubules of other treatment groups remained unchanged (Fig. 4). Germinal epithelial layer in Ta, Tp, Tap and Taf were reduced and, in some cases, fragmented. Luminal area of Ta, Tp, Tap and Taf groups were observed to be increased due to absent of growing germinal cells.

### 3.5 Changes in spermatogenesis and Johnsen’s scoring

**Fig. 5:**
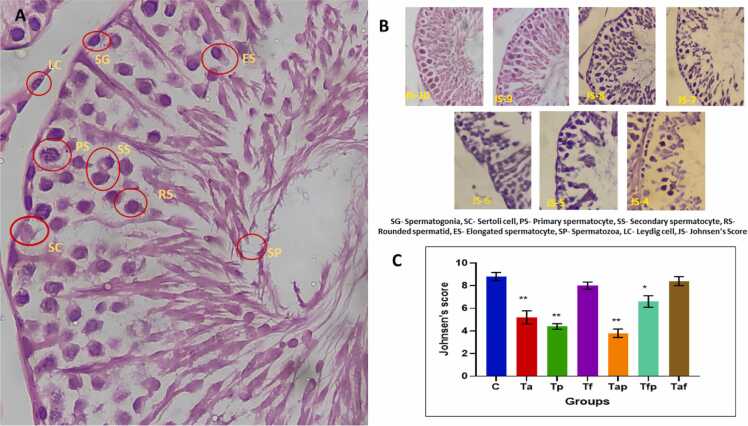
A. Photomicrograph showing different stages of spermatogenic cells. B. Different types of seminiferous tubules matched to different degree of Jonsen’s Score (JS-10 to JS-4). C. Graphical presentation of Jonsen’s score of control group and six treatment groups. The values were expressed as Mean ± SEM (n = 5). * p < 0.05 and **p < 0.01.

Johnsen’s scoring pattern was analysed in all the groups (Fig. 5) according to the criteria of Table 1. High reduction in Jonsen’s score in Ta (p < 0.01), Tp (p < 0.01) and Tap (p < 0.01) groups were seen when compared to the control group. A moderate reduction in Jonsen’s score was found in Tfp (p < 0.05) group. Tf and Taf group exhibited no any significant changes to their Jonsen’s score when compared to that of the control group (Fig. 5).

### 3.6 Effects on cholesterol levels in serum and testicular tissue

**Fig. 6:**
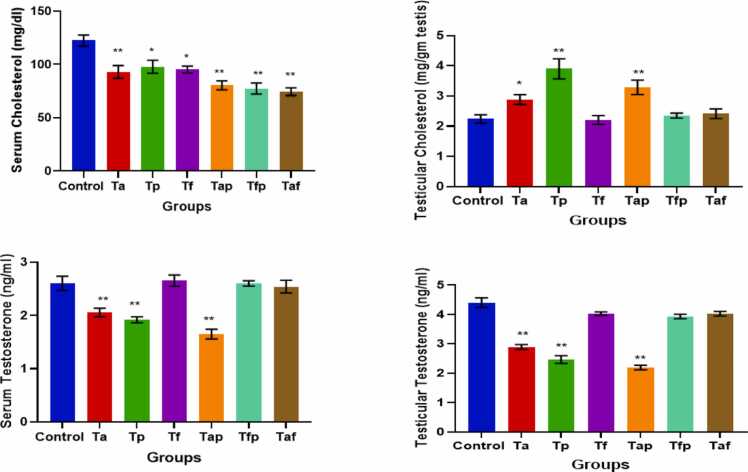
Graphical presentation of Serum cholesterol, Testicular Cholesterol, Serum Testosterone and Testicular Testosterone levels in Control and six treatment groups. The values were expressed as Mean ± SEM (n = 5). * p < 0.05 and **p < 0.01.

Serum cholesterol levels were significantly decreased in all treatment groups (Ta, Tp, Tf, Tap, Tfp and Taf) in comparison with the control group (Fig. 6). On the other hand, cholesterol content in testicular tissues of Ta (p < 0.05), Tp (p < 0.01) and Tap (p < 0.01) groups increased significantly when compared to that of control group (Fig. 5). But testicular cholesterol level in Tf, Tfp and Taf were remained unchanged in comparison with control group (Fig. 6).

### 3.7 Effects on oxidative stress markers in epididymal spermatozoa and testis

**Fig. 7:**
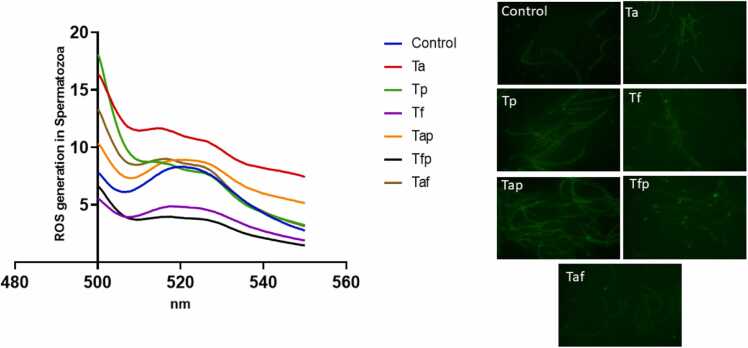
Flowcytometric analysis of ROS generation by DCFDA method in control group and treatment groups.

**Fig. 8:**
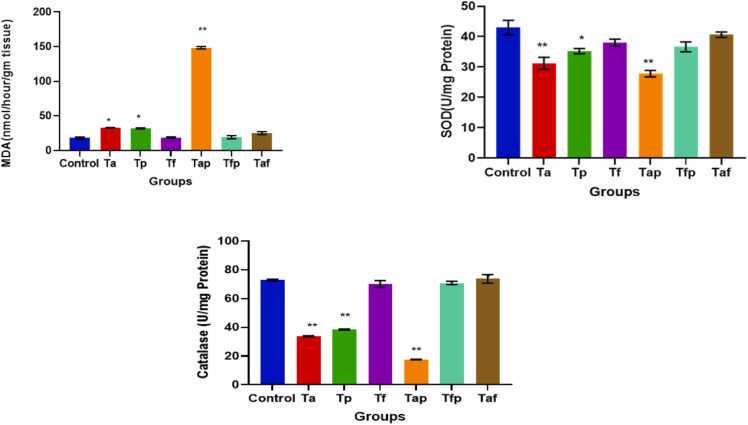
Graphical presentation of MDA, SOD and Catalase activities in all experimental groups. The values were expressed as Mean ± SEM (n = 5). * p < 0.05 and **p < 0.01.

Result of flowcytometry analysis for ROS generation in epididymal spermatozoa of control group and six treatment groups were depicted in Fig. 7. ROS generation enhanced in Ta, Tp, Tap and Taf groups compared to the control group whereas in Tf and Tfp groups amount of ROS decreased (Fig. 7). Results of lipid peroxidation or MDA generation in control group and treatment groups are depicted in Fig. 8. MDA generation had increased significantly in Ta (p < 0.05), Tp (p < 0.05) and Tap (p < 0.01) groups. On the other hand, Tf, Tfp and Taf groups remained unchanged.

### 3.8 Effects on activities of antioxidant enzymes in testis

SOD activities were decreased significantly in Ta (p < 0.01), Tp (p < 0.05) and Tap (p < 0.01) groups and remained unchanged in Tf, Tfp and Taf groups when compared to the control group (Fig. 8). Catalase activities were also decreased significantly in Ta (p < 0.01), Tp (p < 0.01) and Tap (p < 0.01) groups and remained unchanged in Tf, Tfp and Taf groups when compared to the control group (Fig. 8).

### 3.9 Effects on testosterone levels

Serum testosterone was found to be decreased significantly in Ta (p < 0.01), Tp (p < 0.01) and Tap (p < 0.01) groups, whereas, Tf, Tfp and Taf groups showed no significant changes in these parameters when compared to the control group (Fig. 6). Testosterone levels in testicular tissues were found to be decreased significantly in Ta (p < 0.01), Tp (p < 0.01) and Tap (p < 0.01) groups, whereas, Tf, Tfp and Taf groups showed no significant changes in these parameters when compared to the control group (Fig. 6).

## 4 Discussion

Reproductive organs are susceptible to numerous external stressors like heavy metals, xenobiotics, microwaves, nanomaterials, and pharmaceuticals [46], [47]. Alterations in the weight of reproductive organs serve as a significant indicator of reproductive toxicity. Variations in testicular weight may indicate interstitial edema or alterations in the seminiferous tubules. Variations in epididymal weight may suggest edema or inflammation, or they may serve as a sensitive indicator of reduced sperm production [48], [49]. This study examined the weight variations of the testis, epididymis, seminal vesicle, and ventral prostate gland (Table 2) in control group and six treatment groups. The data presented in Table 2 indicate that weight changes primarily occurred in the groups treated with atorvastatin and piperine, as well as in the group co-treated with both atorvastatin and piperine. In contrast, the presence of fenofibrate in the Tf, Tfp, and Taf groups demonstrated minimal to no change in the weight of reproductive organs when compared to the control group (Table 2). Decrease in reproductive organs weight due to the action of atorvastatin and piperine treatments were reported in the previous studies [50], [51] and may be the sign of reproductive toxicity initiated by the activities of atorvastatin and piperine [52]. Fenofibrate at a dose of 20 mg/kg body weight significantly prevented relative weight loss of reproductive organs (testis, epididymis, ventral prostate, and seminal vesicle) when co-administered with atorvastatin and piperine (Table 2) and it implied that fenofibrate might have protective roles over atorvastatin and piperine induced reproductive organ toxicity.

Sperm parameters (Sperm count, viability, and motility) analysis revealed that atorvastatin and piperine caused significant decrease in epididymal sperm count, viability, and motility whereas fenofibrate treatment along with piperine and atorvastatin showed no significant changes compared to the control group (Table 3). These observations implied that fenofibrate prevented the toxic effects of atorvastatin and piperine on sperm parameters.

Exposure to atorvastatin and piperine altered normal histomorphometry of seminiferous tubules whereas animals treated with fenofibrate along with atorvastatin and piperine showed no any remarkable changes in seminiferous tubule diameter, lumen diameter, germinal epithelium height and interstitial space (Table 4). Fenofibrate treatment along with atorvastatin and piperine prevented the distortion of normal histomorphometry of seminiferous tubules.

The advantageous benefits of fenofibrate are substantiated by the amelioration of testicular histological lesions caused by atorvastatin and piperine, as evidenced by elevated Johnson’s scores, indicating their potential protective impact against atorvastatin, and piperine-induced testicular injury. To the best of our knowledge, the efficacy of fenofibrate in protecting relative reproductive weight, sperm count, motility and viability, testosterone levels, and histological changes in the atorvastatin and piperine-induced testicular toxicity paradigm remains unexamined.

Oxidative stress is recognised as a significant factor in testicular damage associated with atorvastatin and piperine induced reproductive toxicity. Atorvastatin and piperine treatment induced excessive reactive oxygen species (ROS) production (Fig. 7) in epididymal spermatozoa, leading to a decrease in antioxidant enzyme levels and an increase in lipid peroxidation within rat testicular tissue (Fig. 8), which is associated with a marked decline in testicular function. In a recent study, Abdel-Aziz et al. [53] demonstrated that fenofibrate mitigates testicular damage induced by streptozotocin in diabetic rats by increasing SOD levels while decreasing MDA levels. Our results also revealed that fenofibrate at a dose of 20 mg/kg body weight along with atorvastatin (8 mg/kg bw) and piperine (10 mg/kg bw) provide protective effects against ROS by elevating catalase activity and decreasing MDA levels in testicular tissues (Fig. 7 & 8). Our findings in this respect are quite similar as revealed by Alqahtani et al. (2023) in the same line of studies on testicular toxicity induced by cisplatin [54].

Spermatogenesis is the key regulator of male reproduction. We used Jonsen’s scoring to evaluate spermatogenic status of control group and all treatment groups. Treatment with atorvastatin and piperine severely affected spermatogenesis but fenofibrate prevented the toxic effects of atorvastatin and piperine when combinedly treated (Table 1 and Fig. 5). Nirupama et al. (2013) elucidated that the reduction of germ cells in seminiferous tubules may result from the impairment of the antioxidant defence system in testicular tissues [55]. Previous research by Malini et al. and D’Cruz et al. indicated that PIP suppressed spermatogenesis in male adult albino rats following 30 days of therapy [21], [22]. Refaie studied the protective role of fenofibrate in testicular ischemia reperfusion [56].

Cholesterol serves as the essential precursor for testosterone biosynthesis within Leydig cells and on the other hand, testosterone plays a crucial role in modulating spermatogenesis and facilitating the emergence of secondary sexual characteristics in males [57]. Our study revealed that testicular cholesterol level increased and testosterone level decreased in atorvastatin, piperine, atorvastatin plus piperine treated groups (Fig. 6). Treatment with fenofibrate along with atorvastatin and piperine showed no significant changes in these parameters (Fig. 6). It can be assumed that testicular cholesterol level increased in atorvastatin, piperine, atorvastatin plus piperine treated groups due to reduction in the use of cholesterol for testosterone production which is reflected in the lowered biosynthesis of testosterone.

Our study revealed that oxidative stress has been recognized as a significant factor in testicular damage resulting from ATR and PIP poisoning. ATR and PIP treatment induces the production of excessive reactive oxygen species (ROS), which diminishes antioxidant enzymes and elevates lipid peroxidation in the testicular tissue of rats, resulting in a notable decline in testicular function. Consequently, the reduction of reactive oxygen species (ROS) is crucial for addressing reproductive harm in patients treated with atorvastatin and piperine.

Fenofibrate is one of the most prevalent PPAR-ɑ agonist, with a highly effective lipid-lowering impact. In the current study rats treated with fenofibrate showed no significant changes in reproductive organs weight compared to the control group and these results were accompanied

by restoration of the normal histopathological patterns of testicular tissue and the spermatogenesis process as compared to control group. The current study indicates that atorvastatin and piperine treated rats co-administered with fenofibrate exhibited a considerable rise in serum testosterone levels, suggesting favourable effects on steroidogenesis and Leydig cell activity. Additionally, fenofibrate restored SOD activities and decreased MDA levels. These actions appear to be linked to the effects dependent on PPARα agonists. We did not find any study in line with our present study. But, fenofibrate treatment ameliorates testicular toxicity in various aspects have been reported by many researches in recent times [53], [54], [56].

The present study revealed that both atorvastatin and piperine treated individually or in combination may have negative impact on sperm parameters, damage testicular microenvironment by causing testicular weight loss and distorting testicular histomorphometry, affected cholesterol and testosterone homeostasis. All these changes were found to be associated with changes in testicular antioxidant defence system and testicular oxidative stress induced either by atorvastatin and piperine individually or by their combined action. Thus, prevention of ROS overproduction is important for the treatment of reproductive damage caused by atorvastatin and piperine. p38 MAPK signaling is engaged in several distinct male reproductive processes and is essential for several cellular activities. Elevated intracellular ROS can activate p38 MAPK, which is significant in the induction of testicular apoptosis [58]. This study presents histopathological findings in the testes of atorvastatin and piperine-treated rats, revealing germinal cell derangement and atrophy with necrosis, which significantly differ from the control group. The spermatogenesis process was significantly suppressed compared to the control group, as indicated by Johnsen’s score, which confirmed structural damage. Thongnuanjan et al. reported that Fenofibrate effectively inhibited the activation of p38 MAPK in testicular tissue and mitigated cisplatin-induced apoptosis [59].

## 5 Conclusion

Findings of the present study suggest that administering Fenofibrate at a dosage of 20 mg/kg/day might protect against testicular damage caused by atorvastatin and piperine through its antioxidant properties. Fenofibrate mitigated testicular damage by maintaining histoarchitecture, enhancing blood testosterone levels, and reinstating sperm count and viability. This work appears to be the first investigation offering novel mechanistic insights into the mechanism of action and potential preventative effects of fenofibrates on testicular cytotoxicity. It may generate new opportunities by presenting a promising medication for male hypolipidemic patients to avert testicular damage.

## Ethics approval

This study was performed in accordance with the guidelines for the care and purpose of laboratory animals. All the experiments were carried out in accordance with the recommendations of the Committee for the Purpose of Control and Supervision of Experiments on Animals (CPCSEA), India (No. 892/GO/ Re/S/01/CPCSEA), with the approval of the Institutional Animal Ethics Committee (IAEC), University of Kalyani.

## Funding

This research did not receive any specific grant from funding agencies in the public, commercial, or not-for-profit sectors.

## CRediT authorship contribution statement

**Maharaj Biswas:** Supervision, Resources, Data curation, Conceptualization. **Sweata Sarkar:** Writing – review & editing, Investigation. **Sanjib Ghosh:** Writing – original draft, Resources, Methodology, Investigation, Formal analysis, Conceptualization.

## Declaration of Competing Interest

The authors declare that they have no known competing financial interests or personal relationships that could have appeared to influence the work reported in this paper.

## Data Availability

Data will be made available on request.
